# Islet Autoantibodies to Pancreatic Insulin-Producing Beta Cells in Adolescent and Adults with Type 1 Diabetes Mellitus: A Cross-Sectional Study

**DOI:** 10.3390/diagnostics13101736

**Published:** 2023-05-14

**Authors:** Khalid Siddiqui, Shaik Sarfaraz Nawaz, Assim A. Alfadda, Muhammad Mujammami

**Affiliations:** 1 Strategic Center for Diabetes Research, College of Medicine, King Saud University, Riyadh 11461, Saudi Arabiaaalfadda@ksu.edu.sa (A.A.A.);; 2 Department of Medicine, College of Medicine & King Saud University Medical City, King Saud University, Riyadh 11461, Saudi Arabia; 3 Obesity Research Center, College of Medicine, King Saud University, Riyadh 11461, Saudi Arabia; 4 University Diabetes Center, King Saud University Medical City, King Saud University, Riyadh 11461, Saudi Arabia

**Keywords:** age, autoantibody, type 1 diabetes, zinc transporter 8, tyrosine-phosphatase-like insulinoma type 2

## Abstract

(1) Background: Type 1 diabetes mellitus (T1D) is a chronic autoimmune disease caused by the destruction of pancreatic insulin-producing beta cells. T1D is one of the most common endocrine and metabolic disorders occurring in children. Autoantibodies against pancreatic insulin-producing beta cells are important immunological and serological markers of T1D. Zinc transporter 8 autoantibody (ZnT8) is a recently identified autoantibody in T1D; however, no data on ZnT8 autoantibody in the Saudi Arabian population have been reported. Thus, we aimed to investigate the prevalence of islet autoantibodies (IA-2 and ZnT8) in adolescents and adults with T1D according to age and disease duration. (2) Methods: In total, 270 patients were enrolled in this cross-sectional study. After meeting the study’s inclusion and exclusion criteria, 108 patients with T1D (50 men and 58 women) were assessed for T1D autoantibody levels. Serum ZnT8 and IA-2 autoantibodies were measured using commercial enzyme-linked immunosorbent assay kits. (3) Results: IA-2 and ZnT8 autoantibodies were present in 67.6% and 54.6% of patients with T1D, respectively. Autoantibody positivity was found in 79.6% of the patients with T1D. Both the IA-2 and ZnT8 autoantibodies were frequently observed in adolescents. The prevalence of IA-2 and ZnT8 autoantibodies in patients with a disease duration < 1 year was 100% and 62.5%, respectively, which declined with an increase in disease duration (*p* < 0.020). Logistic regression analysis revealed a significant relationship between age and autoantibodies (*p* < 0.004). (4) Conclusions: The prevalence of IA-2 and ZnT8 autoantibodies in the Saudi Arabian T1D population appears to be higher in adolescents. The current study also showed that the prevalence of autoantibodies decreased with disease duration and age. IA-2 and ZnT8 autoantibodies are important immunological and serological markers for T1D diagnosis in the Saudi Arabian population.

## 1. Introduction

Type 1 diabetes mellitus (T1D) is a chronic autoimmune disease caused by the destruction of pancreatic insulin-producing beta cells [1,2]. It is characterised by increased blood glucose levels and hyperglycaemia, resulting in severe insulin deficiency owing to loss of insulin-producing beta cells. T1D is one of the most common endocrine and metabolic disorders occurring in childhood or adolescence [1]. As of 2021, over 1.2 million children and adolescents younger than 20 years old worldwide reportedly have T1D, and this number is increasing annually [2]. The age-adjusted incidence of T1D in Saudi Arabian children has been reported to be 31.4 per 100,000 population per annum, with Saudi Arabia ranking the 8th country in the world in terms of the incidence rate [3]. However, data on the incidence and prevalence of T1DM remain limited, and only a few studies have been conducted on the epidemiology of T1D in this region [4].

Although the aetiology of T1D is not completely understood, its pathogenesis is thought to be initiated by specific T-cell-mediated destruction of pancreatic beta cells [1]. B lymphocytes were required for the initiation of T-cell-mediated autoimmune diabetes in a nonobese diabetic (NOD) mouse model [5]. Depletion of B lymphocytes has been shown to slow the progression of diabetes in NOD mice. B lymphocytes play an important role in antigen-presenting cells, exhibiting significant quantities of class II major histocompatibility complex antigens and producing cryptic peptides that T lymphocytes cannot withstand [6]. In NOD mice, the administration of an antibody that targets a human cluster of differentiate 20 (CD20) on b cells lowered the number of b cells and reduced the onset of diabetes [7]. In T1D individuals who have recently developed the disease, rituximab (anti-CD20 monoclonal antibody) treatment decreases the immune-mediated destruction of beta cells and preserves beta cell activity [6].

The process of beta cell death is indicated by the development of beta cell autoantibodies. Autoantibodies against pancreatic beta cells are important immunological, metabolic, and serological markers of T1D [8]. Multiple islet-specific autoantibodies, such as islet cell antigen (ICA), insulin antigen (IAA), glutamic acid decarboxylase 2 (GAD65), tyrosine phosphatase like insulinoma type 2 (IA-2), and zinc transporter (ZnT8), are found on secretory granules within pancreatic beta cells, and these autoantibodies can be measured in circulation from a few weeks to up 20 years before the onset of clinical disease [1]. T1D-associated autoantibodies appear months or years before symptom onset. The likelihood of developing overt diabetes is assumed to be more closely correlated with the quantity of antibodies rather than any particular antibody [9]. In most individuals, the presence of one or multiple islet-specific autoantibodies may progress to symptomatic T1D [1].

The prevalence and frequency of IA-2 and ZnT8 have not been studied in Saudi patients with T1D. Only one study has reported GAD to be the most frequently detected autoantibody in patients who are newly diagnosed with T1D [10]. There is a scarcity of T1D data and data on islet-specific autoantibodies in our community with a high prevalence of diabetes. This study aimed to investigate the prevalence of islet autoantibodies in adolescents and adults with T1D according to age and disease duration. In addition, we determined the utility of IA-2 and ZnT8A as immunologic, metabolic, and serological markers of T1D, either alone or in combination.

## 2. Materials and Methods

### 2.1. Participants

A total of 270 patients were enrolled in this cross-sectional study at the University Diabetes Center, King Saud University Medical City, King Saud University, Saudi Arabia, between August 2019 and August 2020. Patients with type 2 diabetes, maturity-onset diabetes of the young, and autoimmune thyroid disease, who were immune-compromised or immunosuppressive drug users, and missing autoantibody results were excluded. A total of 108 patients (50 male and 58 female) with T1D autoantibody levels were identified and included in this study. Of these 108 patients, 56 were adolescents, and 52 were adults.

This study was reviewed and approved by the Institutional Review Board of the College of Medicine, King Saud University, Riyadh (IRB No. E-19-4192). Informed consent was obtained from all the participants included in the study. The study was carried out in accordance with the code of ethics of the World Medical Association (Declaration of Helsinki).

### 2.2. Type 1 Diabetes Diagnosis

T1D was diagnosed according to the American Diabetes Association criteria [7]: fasting plasma glucose (FPG) ≥ 126 mg/dL (≥7 mmol/L) or 2-h plasma glucose ≥ 200 mg/dL (≥11.1 mmol/L) during an oral glucose tolerance test or glycated haemoglobin A1C ≥ 6.5 (48 mmol/mol), low or undetectable levels of plasma C-peptide, insulin therapy administration at T1D diagnosis in patients with classical symptoms of hyperglycaemia or hyperglycaemic crisis, and random plasma glucose ≥ 200 mg/dL (≥11.1 mmol/L). Patients with T1D typically present with symptoms of polyuria, polydipsia, and ketoacidosis.

### 2.3. Clinical and Laboratory Data

Demographic data such as age, sex, weight, height, duration of disease, and family history of diabetes were collected from the patient files by the research physician. Blood samples were obtained from all the participants and centrifuged to collect the serum on the day of the clinic visit. Serum samples were stored at −20 °C until use. Clinical and laboratory tests, including those for blood glucose, glycated haemoglobin (HbA1c), haemoglobin, alanine aminotransferase (ALT), aspartate aminotransferase (AST), gamma-glutamyl transferase, and alkaline phosphatase, were performed using a biochemistry analyser (Randox Daytona, Randox Laboratories, Crumlin, UK).

### 2.4. Islet Autoantibody Assay

Participants were screened for human IA-2 autoantibody (Catalogue No.: RIAE/96R, BioVendor, Brno, Czech Republic) and ZnT8 autoantibody (Catalogue No.: RZnT8/96R, BioVendor, Brno, Czech Republic) using an enzyme-linked immunosorbent assay kit. The IA-2 and ZnT8 autoantibody concentrations in patient serum were read from the calibration curves constructed in the same run as the calibrators expressed in U/mL. The cut-off values for the IA-2 and ZnT8 autoantibodies were <7.5 U/mL and <15 U/mL, respectively. The intra- and inter-assay precisions of variation for the IA-2 autoantibody were 1.3–4.5% and 3.5–9.3% for the ZnT8 autoantibody, respectively. The IA-2 autoantibody showed 98% specificity and 76% sensitivity, and the ZnT8 autoantibody showed 97% specificity and 76% sensitivity. This information was obtained from the manufacturer’s information sheet.

### 2.5. Statistical Analysis

All the data are expressed as mean ± standard deviation or median (interquartile range) and percentage. We compared the adolescent and adult groups using the Student’s t-test for the continuous data and the chi-square test for the categorical data. The relationship between disease duration and autoantibodies was categorised into tertiles (<1 year, 1–5 years, 6–10 years, and ≥11 years). Similarly, the relationship between age and autoantibodies was categorised into tertiles (<12 years, 13–17 years, 18–22 years, 23–27 years, 28–32 years, and >33 years). Multinomial logistic regression analysis was used to establish the independent predictors of autoantibody status. All the statistical analyses were performed using SPSS version 21 (IBM, Armonk, NY, USA). Statistical significance was set at *p* ≤ 0.05.

## 3. Results

The baseline characteristics of the selected 108 patients with T1D are shown in Table 1. Of these 108 patients, 51.8% were adolescents, and 48.2% were adults. The mean age of the study population was 17.0 years; 53.7% were female, and 46.3% were male. A total of 48.1% of patients had a family history of diabetes. IA-2 autoantibody positivity was highest and found in 67.6% of patients with T1D, while ZnT8 positivity was detected in 54.6% of patients. Our biochemical results showed that the adolescents demonstrated higher glucose concentration, higher HbA1c levels, and higher alkaline phosphate compared to adults. Our results showed that the prevalence of IA-2 autoantibody in adolescents was higher than that in adults. Similarly, our results showed that the prevalence of ZnT8 autoantibody in adolescents was higher compared to that of adults; however, the findings were not significant. Adolescents demonstrated higher antibody positivity compared to adults.

A total of 86 autoantibody positive individuals were included in the analysis, divided into those with a single autoantibody (*n* = 40) and multiple autoantibodies (*n* = 46) (Table 2). There was a similar proportion of male participants in those with single and multiple autoantibodies. IA-2 and ZnT8 autoantibodies were significantly higher in the multiple autoantibody group compared to the single autoantibody group. There was no significant difference in the family history, HbA1c, and blood glucose levels between the single and multiple autoantibody groups.

In our study, patients with T1D exhibited islet autoantibody in relation to the disease duration. The pattern of positive IA-2 and ZnT8 autoantibodies varied among the four disease duration groups. The proportion of patients with positive IA-2 decreased with increasing disease duration. Moreover, the proportion of patients with positive ZnT8 decreased with increasing disease duration as shown in Table 3.

**Figure 1:**
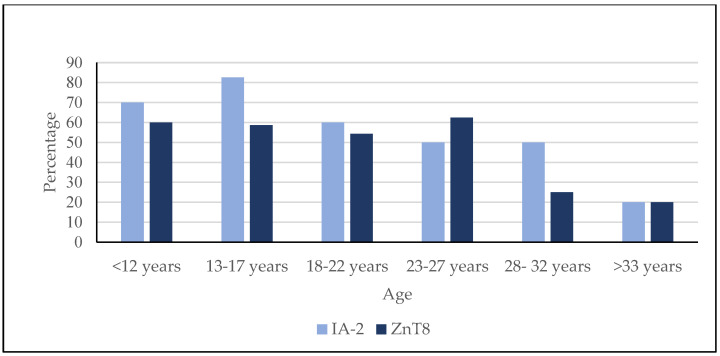
Type 1 diabetes patients exhibit islet autoantibody in relation to age. The prevalence of IA-2 saw a significant decrease relating to age, while the prevalence of ZnT8 also decrease with age but was not significant.

In our study, T1D exhibited islet autoantibody in relation to age as shown in Figure 1. The pattern of positive IA-2 and ZnT8 autoantibodies varied among the six age groups. The proportion of patients with positive IA-2 decreased with increasing age. Conversely, the proportion of patients with positive ZnT8 antibody decreased with increasing age, as shown in Table 4.

Our results also showed that age is an independent predictor of autoantibody status, while sex, family history of diabetes, disease duration, and HbA1c did not affect the autoantibody status (Table 5).

## 4. Discussion

In the current study, we demonstrated that adolescents with T1D had higher rates of IA-2 and ZnT8 autoantibodies compared to adults. According to age and disease duration, the prevalence of IA-2 and ZnT8 autoantibodies decreased. Age and autoantibody levels were found to significantly interact with each other.

Only a few studies conducted in Saudi Arabia [11], the UAE [12], Sudan [13], Iran [14], and Qatar [15] have reported GAD to be the most commonly detected autoantibody in patients with T1D in Arab countries. Most available studies have not evaluated the utility of the ZnT8 autoantibody in the diagnosis and prediction of T1D. T1D autoantibody data in communities with a high prevalence of diabetes, such as ours, are limited. The islet autoantibody distribution varies by age, and screening strategies for T1D with different onset ages are lacking [16]. Therefore, examining IA-2 and ZnT8 autoantibodies according to age and disease duration can add to the knowledge on the clinical manifestations of T1D.

IA-2 is a transmembrane glycoprotein of the tyrosine-phosphatase-like protein family, and ZnT8A, the product of the *SLC30A8* gene located on chromosome 8, is localised to the insulin secretory granules of beta cells [17,18]. Variations in *SLC30A8* may affect zinc accumulation in beta cells (insulin granules) and insulin processing, crystallisation, and secretion [19]. IA-2 and ZnT8 are major autoantibodies involved in the development of autoimmunity in T1D [19]. The prevalence and autoantibody titres of ZnT8, GAD-65, and IA-2 decline significantly with disease progression and increased duration of diabetes in Latin Americans [20]. A study from Sudan reported that 69.7% of adolescents with T1D are autoantibody positive [13]. Another study from Iran also reported that 81.7% of adolescents with T1D were autoantibody positive [14]. In our study, we also revealed (Table 1) that autoantibody positivity was more common in adolescents at younger ages (91.1%) compared to adults (67.3%). Conversely, in a Pittsburgh study on epidemiology of diabetes complications, older age at the onset of childhood T1D was associated with autoantibody positivity [21].

Elmaoğulları S et al. reported the prevalence of ZnT8 autoantibodies in Turkish adolescents to be 58% [22]. Thewjitcharoen et al. also assessed the beta cell function with the status of islet autoantibody in long-standing Thai patients with T1D [23]. The prevalence rates of GAD, IA2, and ZnT8 autoantibodies were reported to be 65%, 20%, and 10%, respectively [23]. Fabris et al. (2015) also reported ZnT8 as a complement to the current T1D autoantibodies (GADA, IA-2A, IAA, and ICA) in a large paediatric Italian population; 49.8% of the patients with T1D were found to be ZnT8 autoantibody positive, confirming ZnT8A as an important additional independent diagnostic marker [24]. Consistent with other studies, in our study, the prevalence of IA-2 autoantibodies in adolescents was reported to be 80.4% compared to adults. The prevalence of ZnT8 autoantibodies in adolescents was higher at 58.9% compared to adults.

Recent data from Poland indicate that the ZnT8 autoantibody is related to the age and metabolic state of patients newly diagnosed with T1D. The prevalence of ZnT8, and IA-2 autoantibodies in children was reported to be 81.1% and 80.7%, respectively, while, in adults, the prevalence was 34.7% [25]. In our study, adolescents with T1D had a higher IA-2 autoantibody titre than adults. Moreover, adolescents with T1D had higher ZnT8 autoantibody titres. Similarly, Niechciał, E et al. demonstrated that adolescents with T1D had significantly higher ZnT8 autoantibody titres, while adults with T1D had higher IA-2 autoantibody titres [25]. 

In 2021, Kawasaki et al. showed that both IA-2 and ZnT8 are frequent in young Japanese patients. The prevalence of IA-2, and ZnT8, decline according to the disease duration. A significant interaction was observed between the age of onset and duration of diabetes, with an onset age of ≤10 years in relation to the IA-2 and ZnT8 autoantibodies. The prevalence of IA-2 and ZnT8 with a duration of diabetes ≤ 3 years was 41.1% and 36.7%, respectively, with 80.0% expressing one or more of these autoantibodies [26].

Petruzelkova et al. assessed the prevalence of ZnT8 in Czech children with newly diagnosed T1D and studied the dynamic changes in the ZnT8 autoantibody levels during disease progression. ZnT8 autoantibodies were detected in 72% of the children with newly diagnosed T1D. ZnT8 autoantibody levels decreased over time following T1D onset, and the levels of ZnT8 autoantibodies correlated with those of the IA-2 autoantibodies [27]. Our results are consistent with those of previous studies [26,27]. In our study, the pattern of positive IA-2 and ZnT8 autoantibodies varied among the four disease duration groups. The proportion of patients with positive IA-2 and ZnT8 decreased with increasing duration of diabetes.

In the Colorado Diabetes Autoimmunity Study in the Young (DAISY), the Finnish T1D prediction and prevention (DIPP), and the German BABYDIAB and BABYDIET studies, the rate of T1D progression in 585 children who developed multiple islet autoantibodies was evaluated. The progression rates of T1D following seroconversion were 43.5%, 69.7%, and 84.2% at 5, 10, and 15 years of follow-up, respectively [28]. The risk of progression to T1D is highly variable and varies according to age, autoantibody type, and metabolic status in the TrialNet PTP individuals with multiple autoantibodies [29]. Fabris et al. (2015) reported ZnT8 autoantibodies as additional and independent diagnostic markers in a large cohort of paediatric Italian patients with T1D [24].

Nicholas et al. used a T1D genetic risk score in patients with clinically suspected T1D to confirm T1D. Furthermore, they evaluated the prevalence and pattern of islet autoantibodies (GAD, IA2, and ZnT8) at diagnosis. The prevalence of islet autoantibodies at diagnosis in genetically confirmed older-adult T1D was the same as that of childhood-onset T1D. This study also suggests that there is no difference in the prevalence of positive islet autoantibodies between the age groups. The pattern of positive islet autoantibodies in genetically consistent T1D cases varied among the three age groups. The proportion of patients with positive GAD increased with the increasing age of diagnosis. Conversely, the proportion of positive IA2 and ZnT8 reduced with increasing age at diagnosis [30].

In our study, the pattern of positive IA-2 and ZnT8 autoantibodies varied among the six age groups. The proportion of patients with positive IA-2 decreased with increasing age. Conversely, the proportion of patients with positive ZnT8 antibodies decreased with age. Our study confirmed similar autoantibody patterns among different age groups [28,30]. Furthermore, Głowińska–Olszewska et al. demonstrated a pattern positivity of IA-2 and ZnT8 decrease with disease duration < 5 years, 6–10 years, and >10 years in Polish patients with T1D. The proportion of patients with IA-2 with disease duration was 59%, 41.0%, and 10.0%, whereas the proportion of patients with ZnT8 with disease duration was 72.0%, 58.0%, and 40.0%, respectively [31].

The differences in the prevalence of islet-specific autoantibodies among patients with T1D in different ethnic populations may be explained by human leukocyte antigen (HLA) haplotypes [28]. HLA haplotype patterns, such as HLA-DR3, DQA1*05:01, DQB1*02:01, and DRB1*03:01 alleles and their haplotypes are associated with the presence of autoantibodies and T1D in the Bahraini, Kuwaiti, Egyptian, and Tunisian populations, while HLA-DR4, DQA1*03:01, DQB1*03:02, and DRB1*04:05 are associated with the presence of autoantibody and T1D in the Saudi Arabian and Algerian population [32,33]. Patients with newly diagnosed T1D carrying the DR4-DQB1*0302 haplotype showed increased levels of IA-2A autoantibodies, and those with the DR3-DQB1*02 haplotype showed increased levels of GADA autoantibodies [33]. In contrast, ZnT8 autoantibody positivity has been correlated with DQ8 haplotypes [34]. IA2 autoantibody positivity has been related to DQ8 haplotypes [32,34]. Taken together, these pieces of evidence suggest that HLA haplotypes have a strong impact on the appearance of islet-specific autoantibody in T1D-affected children and in the general population.

Our study had a few limitations. First, it was a small study conducted in a single centre in Riyadh. Second, our study participants had variable age and disease durations. Third, we did not examine the autoantibody cut-offs in the general population. Fourth, we did not examine islet-specific autoantibodies such as GAD65 and ICA. Lastly, we did not examine the relationship between islet autoantibodies and genetic factors, especially HLA genotypes.

## 5. Conclusions

Screening for IA-2 and ZnT8 autoantibodies alone identifies the vast majority of T1D patients. T1D adolescents reported greater levels of IA-2 and ZnT8 autoantibodies. The prevalence of IA-2 and ZnT8 autoantibodies decreased with age and disease duration. Age and autoantibody levels were discovered to have substantial interaction. Larger prospective multicentre studies among different ethnicities in Arab populations are warranted.

## Figures and Tables

**Table 1:** The clinical, biochemical, and prevalence of ZnT8, and IA-2 autoantibodies in adolescent and adult T1D patients.

Variables	Total Subject (*n* = 108)	Adolescent (*n* = 56)	Adults (*n* = 52)	*p*-Value
Age (years) [IQR]	17.0 (14.0–20.0)	14.5 (13.0–16.75)	20.5 (19.0–25.0)	0.001 *
Sex, *n* (%)				
Female *n* (%)	58 (53.7)	32 (57.1)	26 (50.0)	0.457
Male *n* (%)	50 (46.3)	24 (42.9)	26 (50.0)	0.457
Family History of Diabetes *n* (%)	52 (48.1)	26 (46.4)	26 (50.0)	0.711
Height (cm)	159.9 ±10.7	156.3 ± 12.1	163.9 ± 6.9	0.001 *
Weight (kg)	59.1 ± 14.5	54.4 ± 15.8	64.3 ± 11.0	0.001 *
Hemoglobulin (g/dL)	13.7 ± 1.6	13.8 ± 1.2	13.6 ± 2.0	0.505
Blood glucose (mmol/L)	11.4 (7.2–16.2)	14.6 (8.5–18.9)	8.7 (5.4–14.3)	0.001
HbA1c mmol/mol	81.0 (66.0–99.5)	93.0 (75.0–107.0)	73.0 (60.0–85.2)	0.001 *
HbA1c (%) (IQR)	9.7(8.2–11.3)	10.7 (9.0–12.0)	8.8 (7.6–9.9)	0.001 *
Alkaline phosphate (U/L)	126.0 (87.5–219.5)	177.5 (116.0–280.7)	90.0 (78.0–129.0)	0.001 *
ALT (U/L) (IQR)	12.6 (10.0–15.9)	13.0 (11.3–15.9)	12.1 (9.2–16.2)	0.881
AST (U/L)(IQR)	16.4 (14.1–19.4)	16.3 (14.1–19.4)	16.4 (14.3–19.6)	0.651
GTT (U/L) (IQR)	14.0 (11.0–20.0)	13.0 (11.0–17.4)	15.5 (12.4–25.4)	0.058
IA-2, autoantibody *n* (%)	73 (67.6)	45 (80.4)	28 (53.8)	0.003 *
IA-2-antibody titer (U/mL)	35.69 (0.1–149.51)	55.46 (13.22–189.18)	15.59 (0.1–97.52)	0.732
Znt8 autoantibody, *n* (%)	59 (54.6)	33 (58.9)	26 (50.0)	0.352
ZnT8-antibody titer (U/mL)	20.1 (6.65–96.17)	26.67(6.69–104.27)	16.33 (6.65–92.39)	0.282
Autoantibody positivity *n* (%)	86 (79.6)	51 (91.1)	35 (67.3)	0.002 *
Number of positive autoantibodies				
1 antibody (IA-2/ZnT8A) (%)	40 (37.0)	24 (42.9)	16(30.8)	0.194
2 antibodies (IA-2+ZnT8A) (%)	46 (42.6)	27 (48.2)	19(36.5)	0.220
Individuals positive for single autoantibody				
IA-2, autoantibody (%)	27 (25.0)	18 (32.1)	9 (17.3)	0.075
Znt8, autoantibody (%)	13 (12.0)	6 (10.7)	7 (13.5)	0.661

Data are expressed as *n* (%), mean ± SD, and median (IQR: interquartile range (25–75th percentile). Abbreviations: HbA1c: Glycated hemoglobin; AST: Aspartate aminotransferase; ALT: Alanine amino transferase; GGT: Gamma-glutamyl transferase; IA-2 autoantibody: tyrosine phosphatase like insulinoma type 2; ZnT8: Zinc transporter 8 autoantibody; * *p* < 0.05 is considered statistically significant.

**Table 2:** Baseline characteristics of autoantibody-positive participants.

Variables	Single Ab+ (*n* = 40)	Multiple Ab+ (*n* = 46)	*p*-Value
Age (years)	17.0 (14.0–18.0)	17.0 (14.0–19.0)	0.510
Sex, *n* (%)			
Female *n* (%)	23 (57.5)	25 (54.3)	0.769
Male *n* (%)	17 (42.5)	21 (45.7)	
Family History of Diabetes *n* (%)	18 (45.0)	20 (43.5)	0.887
Hemoglobulin (g/dL)	13.7 ± 1.6	13.5 ± 1.5	0.484
Blood glucose (mmol/L) (IQR)	11.0 (6.6–16.0)	13.3 (7.1–17.3)	0.406
HbA1c mmol/mol (IQR)	80.5 (67.5–99.2)	85.0 (61.5–102.0)	0.787
HbA1c (%) (IQR)	9.5 (8.3–11.2)	9.9 (7.8–11.7)	0.599
IA-2 autoantibody, *n* (%)	27 (67.5)	46 (100)	0.001 *
Znt8 autoantibody, *n* (%)	13 (32.5)	46 (100)	0.001 *

Data are expressed as *n* (%), mean ± SD, and median (IQR) (25–75th percentile). Abbreviations: Ab+: Antibody positive; HbA1c: Glycated hemoglobin IA-2: insulinoma-associated antigen 2; ZnT8: Zinc transporter 8; Single Ab+: Single autoantibody positive; Multiple Ab+: Multiple autoantibody positive. * *p* < 0.05 is considered statistically significant.

**Table 3:** The relationship between disease duration and the prevalence of IA-2 and ZnT8 autoantibodies.

Autoantibodies	Disease Duration				*p*-Value
	<1 year	1–5 year	6–10 year	≥11 year	
IA-2 *n* (%)	8 (100)	24 (77.4)	21 (67.7)	17 (51.5)	0.027 *
ZnT8 *n* (%)	5 (62.5)	14 (45.2)	24 (77.4)	14 (24.6)	0.020 *
IA-2/ZnT8 Positive *n* (%)	3 (37.5)	14 (45.2)	13 (41.9)	9 (27.3)	0.476
IA-2+ZnT8 Positive *n* (%)	5 (62.5)	12 (38.7)	16 (51.6)	11 (33.3)	0.299

Data are expressed as *n* (%). Abbreviations: IA-2: insulinoma-associated antigen 2; ZnT8: Zinc transporter 8; * *p* < 0.05 is considered statistically significant.

**Table 4:** The relationship between age and the prevalence of IA-2 and ZnT8 autoantibodies.

Autoantibodies	Age Groups						*p*-Value
	<12 years	13–17 years	18–22 years	23–27 years	28–32 years	>33 years	
IA-2 *n* (%)	7 (70.0)	38 (82.6)	21 (60.0)	4 (50.0)	2 (50.0)	1 (20.0)	0.028 *
ZnT8 *n* (%)	6 (60.0)	27 (58.7)	19 (54.3)	5 (62.5)	1 (25.0)	1(20.0)	0.485
IA-2/ZnT8 Positive *n* (%)	3 (30.0)	21 (45.7)	14 (40.0)	1 (12.5)	1(25.0)	1(0)	0.216
IA-2+ZnT8 Positive *n* (%)	5 (50.0)	22 (47.8)	13 (37.1)	4 (50.0)	1 (25.0)	1 (20.0)	0.716

Data are expressed as *n* (%). Abbreviations: IA-2: insulinoma-associated antigen 2; ZnT8: Zinc transporter 8; * *p* < 0.05 is considered statistically significant.

**Table 5:** Multinomial logistic regression analysis was used to establish the independence of predictors of autoantibody status.

Variables	B	Std. Error	Wald	Exp (B)	95% Confidence Interval for Exp (B)		*p*-Value
					Lower Bound	Upper Bound	
Intercept	3.688	2.254	2.676				0.102
Age in years	−0.156	0.054	8.245	0.855	0.769	0.952	0.004 *
Gender (1 = Male; 2 = Female)	0.457	0.574	0.634	1.580	0.513	4.869	0.426
Family History of Diabetes (1 = No; 2 = Yes)	−0.452	0.575	0.618	0.636	0.206	1.965	0.432
Disease duration (1 = <5 years; 2 = >5 years)	0.243	0.685	0.126	1.275	0.333	4.869	0.723
HbA1c %	−0.003	0.135	0.000	0.982	0.997	0.765	1.299

* *p* < 0.05 is considered statistically significant.

## Data Availability

Data will be made available upon appropriate request.
